# Accreditation, setting and experience as indicators to assure quality in oncology biomarker testing laboratories

**DOI:** 10.1038/s41416-018-0204-9

**Published:** 2018-08-24

**Authors:** Véronique Tack, Ed Schuuring, Cleo Keppens, Nils ‘t Hart, Patrick Pauwels, Han van Krieken, Elisabeth M.C. Dequeker

**Affiliations:** 1 Biomedical Quality Assurance Research Unit, Department of Public Health and Primary Care, KU Leuven, Kapucijnenvoer 35, 3000 Leuven, Belgium; 2 Department of Pathology, University of Groningen, University Medical Center Groningen, Hanzeplein 1, PO Box 30001, 9700 RB Groningen, The Netherlands; 3 Center for Oncological Research (CORE), University of Antwerp, Universiteitsplein 1, 2610 Wilrijk, Belgium; 4 Department of Pathology, Radboud University Medical Center, Geert Grooteplein zuid 10, Huispost 824, 6525 Nijmegen, The Netherlands

**Keywords:** Biomarkers, Cancer

## Abstract

**Background:**

Predictive biomarkers allow clinicians to optimise cancer treatment decisions. Therefore, molecular biomarker test results need to be accurate and swiftly available. To ensure quality of oncology biomarker testing, external quality assessments (EQA) for somatic variant analyses were organised. This study hypothesised whether laboratory characteristics influence the performance of laboratories and whether these can be imposed before authorisation of biomarker testing.

**Methods:**

Longitudinal EQA data from the European Society of Pathology were available over six (metastatic colorectal cancer) and four years (non-small cell lung cancer), including the percentage of analysis errors and technical failures, and information on laboratory characteristics (accreditation status, laboratory setting, number of samples analysed and detection method). Statistical models for repeated measurements were used to analyse the association between the EQA results and the laboratory characteristics.

**Results:**

Laboratory accreditation was associated with fewer analysis errors in early stages of biomarker introduction into the laboratory. Analysing more samples, or university and research laboratories showed better performance. Changing the detection method did not have an effect.

**Conclusion:**

The indicators support the clinicians in choosing molecular pathology laboratories by improving quality assurance and guaranteeing patient safety. Accreditation of laboratories, centralisation of biomarker testing or a university and research setting should be stimulated.

## Introduction

Pathology laboratories are challenged to maintain high quality assurance due to the constant pressure of newly developed technical and medical expertise. Research on predictive biomarkers has gained considerable momentum, highlighting the need for regular updates of test strategies.^1,2^

Predictive biomarkers allow clinicians to predict clinical effects of cancer treatments.^3^ For this purpose, biomarker test results need to be very accurate and swiftly available. For metastatic colorectal cancer (mCRC), the confirmation of a wild-type *KRAS* and *NRAS* gene is required before anti-EGFR monoclonal antibody therapies.^4–7^ In the context of non-small-cell lung cancer (NSCLC), treatment with EGFR tyrosine kinase inhibitors requires the presence of an activating variant in the *EGFR* gene. Moreover, *ALK* and *ROS1* gene tyrosine kinase inhibitor (TKI) treatment can only be administered to patients with a confirmed gene rearrangement in the mentioned genes.^8–11^ Recently, the immune checkpoint inhibitor, pembrolizumab, was approved by the US Food and Drug Administration (FDA) in first line therapy for patients with ≥50% positive PD-L1 expression in NSCLC, as assessed by immunohistochemistry (IHC).^12^ The immune checkpoint inhibitor nivolumab can be given to patients with NSCLC without confirmation of PD-L1 positivity.

To ensure quality of oncology biomarker testing, external quality assessments (EQA) are organised by the European Society of Pathology (ESP) according to international standards.^13^ Remarkably, each year a significant number of laboratories are not able to correctly identify the relevant variants.^14,15^

Previous research by our group in 2014 demonstrated that laboratories were not able to rapidly introduce new accurate biomarker testing for the use of cetuximab and panitumumab, two anti-EGFR therapies for patients with mCRC.^14^ On the other hand, multiple participations in EQA programs showed improved performance rates.^15,16^ However, the number of genotyping errors remains high.^14,15^ Laboratories are also challenged by the large variation in mutation types, for example targeted variants in the *EGFR* gene include point mutations, insertions and deletions, which evolve over time.^17^ With the large number of ongoing phase-III clinical trials, and the rapid developments in biomarker testing, several new testing strategies and relevant biomarkers are expected to be implemented each year.^1,18^ The insufficient performance of laboratories in EQA together with the increasing number of biomarker tests are subjects of concern. Laboratories should be stimulated to maintain a high degree of quality assurance by means of continuous education and development of expertise in oncology biomarkers. Several longitudinal studies were already performed on data from EQA in oncology biomarker testing, but the influence of specific laboratory characteristics on the performance of oncology biomarker testing has, to date, not yet been analysed.^19^

Laboratory characteristics, such as accreditation, experience and laboratory setting or organisational structure, are recognised as important elements that could influence the performance of laboratories.^20–22^ How can the hypothesis be justified that accreditation or a minimal level of experience can be imposed before authorisation of biomarker testing?

The aim of this study was to correlate laboratory characteristics with EQA results in biomarker testing for NSCLC and mCRC in order to identify important quality indicators.

## Materials and methods

The EQA schemes of the ESP were coordinated by the Biomedical Quality Assurance (BQA) Research Unit of the KU Leuven according to international guidelines.^13,23^ The schemes were open to all laboratories worldwide (Table 1). The participants had to report their results in an online datasheet, together with questions on laboratory characteristics and testing strategies. It was mentioned in this datasheet that data could be used for further research. In addition, mock diagnostic reports were requested based on the information provided by the BQA Unit of the KU Leuven. Medical and technical experts assessed the data provided by the participants in collaboration with the EQA coordination centre. A laboratory received full marks (2 points) when the genotype of the requested biomarker was correctly assigned; points were deducted in case of clerical errors, technical failures, analysis errors or nomenclature errors. Each sample result was evaluated by two assessors independently, and equivocal results were discussed during a meeting. Samples for which more than 25% of the laboratories could not obtain a reliable result were not considered for the analyses.^24^ These were defined as educational samples.

**Table 1:** Overview of the samples and number of participants included in each EQA

EQA scheme	Scheme year	Number of samples distributed	Number of participants	Number of successful laboratories (≥90%)	Percentage analysis errors	Percentage technical errors
Colon (*KRAS/NRAS*)	2010	10	103	598	4.3%	1.4%
	2011	10	124			
	2012	10	105			
	2013	10	131			
	2014	10	125			
	2016	10	123			
*EGFR*	2013	4	106	280	8.7%	5.0%
	2014	9	144			
	2015	9	114			
	2016	10	97			
*ALK* FISH	2012	5	54	379	3.2%	4.3%
	2013	5	100			
	2014	8	116			
	2015	10	111			
ALK IHC	2012	8	29	296	4.8%	1.2%
	2013	12	48			
	2014	9	96			
	2015	5	95			
*ROS1* FISH	2014	8	56	92	2.6%	7.4%
	2015	9	68			
ROS1 IHC	2014	10	31	42	8.9%	0.2%
	2015	5	31			

Educational cases were excluded, as they were not taken into account to determine the EQA score

*EQA* external quality assessment

The EQA schemes of the ESP can be categorised into two groups: those for variant analysis (*RAS genes*, including *KRAS* and *NRAS*, in mCRC and the *EGFR* gene in NSCLC) and those for gene rearrangement and expression analyses (*ALK* and *ROS1* rearrangements in NSCLC).

For variant analyses, participants should use their routine procedures to analyse the EQA samples. The sent samples were formalin-fixed paraffin-embedded (FFPE) material from the resection specimens, commercial FFPE reference standards (Horizon Diagnostics, Cambridge, U.K., http://www.horizondx.com and Thermo Fisher Scientific; Fremont, California, www.thermoscientific.com/qc) or cell line material.^19^ The cell line material was chosen as a pilot for standardisation of homogeneous samples for testing. The percentages of neoplastic cells varied between 10 and 100%.

For gene rearrangement and expression analyses, both IHC and fluorescent in situ hybridisation (FISH) techniques were included in the EQA schemes. The samples included FFPE cell lines on glass slides as well as tissue microarrays from several FFPE blocks from either the cell line material or the resection specimen.^19^

The association study included the EQA results as the dependent variable and the laboratory characteristic as the independent variable (Table 2). For the EQA results, the total EQA scores were taken into account, but also, the isolated percentage of analysis errors or the number of technical failures were considered. Other specific error types were not analysed individually, but remain integrated in the EQA scores. Analysis errors consisted of false positive (reported a variant, rearrangement or aberrant expression in a wild-type sample), false negative (wildtype was reported in a tumour containing a variant, rearrangement or aberrant expression) or wrongly reported variants (a variant was found, but it was an incorrect variant). Technical failures arose when no conclusive results were reported by the participant, either due to a limited amount of DNA recovered from the sample or a doubtful result.

**Table 2:** Investigated characteristics, including the subgroups, and the number of observations for each EQA scheme

Characteristics	Subcategories of the characteristics	Colon EQA scheme 2010–2016 (all laboratories)	Colon EQA scheme 2013–2016 (*RAS* testing laboratory^a^)	Lung *EGFR* EQA scheme 2012–2016	Lung *ALK* EQA scheme 2012–2016	Lung *ROS1* EQA scheme 2014–2015
Number of observations with the characteristic						
Accreditation	Gene-specific accreditation	94	23	44	59	5
	Non-specific accreditation	143	114	107	128	49
	No accreditation	454	210	302	383	82
	Missing data	20	1	8	17	0
Setting	Industry^b^	22	14	16	9	3
	(Private) laboratories^c^	108	63	76	55	13
	Hospital laboratories^d^	238	104	121	104	25
	University and research^e^	343	167	248	326	83
	Missing data	0	0	0	0	0
Number of samples	<10	33	4	23	49	39
	10–99	174	69	104	120	50
	100–249	231	137	148	115	18
	250–499	160	81	124	73	11
	≥500	90	42	58	27	4
	Missing data	23	11	4	6	2
Methods	Non-NGS-based commercial methods				/	/
	Non-NGS-based laboratory-developed methods		See Fig. 2			
	NGS-based methods					

Each observation is a participation of a specific laboratory in a specific EQA scheme. For the accreditation status, different national and international standards were taken into account: ISO 15189 and ISO 17025 standards as recognised international accreditation standards,^25,26^ CAP 15189 (College of American Pathologists) as national accreditation standard and widely used national standards such as the national standard in the Netherlands (CCKL) and the standards of the Clinical Pathology Accreditation in the United Kingdom^27–30^

*EQA* external quality assessment, / information not present, *NGS* next-generation sequencing

^a^RAS testing laboratories included those laboratories that tested KRAS and NRAS genes

^b^Laboratories involved in the development of diagnostic commercial kits

^c^Laboratories that are not present within a hospital's infrastructure

^d^Hospital laboratories included private and public hospital laboratories

^e^This setting included education and research hospitals, university hospitals, university laboratories and anti-cancer centres

Four laboratory characteristics were used for association with EQA results of the laboratories, namely the accreditation status of a laboratory, their setting, the number of samples tested each year and the change in used methods. The corresponding subcategories and number of observations per characteristic can be found in Table 2. Each observation of a characteristic denotes the participation of a specific laboratory in a specific EQA scheme. For the accreditation status, different national and international standards were considered. The ISO 15189 and the ISO 17025 standards were included as recognised international accreditation standards.^25,26^ Several national accreditation standards, such as CAP 15189 (College of American Pathologists), and some widely used national standards were also taken into account for accreditation. Examples of the latter are the national standard in the Netherlands (CCKL) and the standards of the Clinical Pathology Accreditation in the United Kingdom.^27–30^ For good readability, both recognised accreditation standards and their equivalents are hereafter referred to as ‘accreditation’. No distinction was made between accreditation standards and their equivalents for further analysis. The reported accreditation status was retrieved from the websites of the relevant national accreditation body. As there is still a lot of confusion in laboratories about accreditation status, these were verified retrospectively.^20^ In case no archived data were available, these data were defined as ‘missing data’ in Table 2. Also, the effect of the setting of a laboratory was studied. The reported setting of the laboratories in the EQA questionnaires was verified on the laboratory’s website. Different categories were assigned and this terminology will be used throughout the manuscript (Table 2). Industry denotes those laboratories involved in the development of diagnostic commercial kits. Private laboratories are laboratories that are not present within a hospital's infrastructure. Hospital laboratories included private and public hospital laboratories. The setting defined as ‘university and research’ included education and research hospitals, university hospitals, university laboratories and anti-cancer centres, all with a clinical demand for biomarker testing. The number of samples tested each year and the used methods were requested during EQA participation and were not further validated. As not all participants provided information on the number of samples, there are some missing data for this characteristic too. Examples of commercial methods (excluding commercial methods based on next-generation sequencing (NGS)) were the Therascreen Pyro Kit (Qiagen), the RAS mutation analysis kit (EntroGen) or the Idylla Mutation Test (Biocartis). Non-NGS-based laboratory-developed techniques (LDT) included Sanger sequencing or TaqMan allelic discrimination assays (Fig. 2).

Longitudinal data between 2010 and 2016 were analysed using statistical models for repeated measurements. Because of the skewed distributions of the EQA scores, a binary outcome was used: <90% (unsuccessful participation) and ≥90% (successful participation). In case an EQA scheme only contained nine samples that were eligible for scoring, because of an educational sample, ≥88.9% was also considered as a successful participation. For binary outcomes (successful participation versus unsuccessful participation), generalised estimating equations (GEE) were used to account for clustering. For continuous outcomes (percentage of analysis errors or technical failures), an unstructured residual covariance matrix was modelled to account for clustering.

All tests were two-sided and a *p*-value of <0.050 was considered significant. Pairwise comparisons were only performed in case of significant results. Analyses were performed with SAS software for Windows (version 9.4).

## Results

This study investigated the hypothesis that whether laboratories would have to comply to specific laboratory characteristics before the authorisation of biomarker testing. Three factors, namely, the accreditation status of a laboratory, their setting and their experience with oncology biomarker testing in mCRC and NSCLC were considered. The latter included the number of samples tested each year and the change in used methods.

### Accreditation

Based on a total dataset of 691 observations (2010–2016) for variant testing in mCRC, no association was found between the accreditation status of laboratories and their EQA score for variant analysis in *KRAS* or *NRAS* genes (Table 3). However, there was a positive effect of accreditation on the percentage of analysis errors for the *NRAS* biomarker (incidence rate ratio (IRR) = 0.52, *p* = 0.023) in the first years of *NRAS* implementation in the laboratory (2013–2014). Pairwise comparisons of different accreditation categories in this group showed that accredited laboratories have 47% fewer analysis errors compared with laboratories without accreditation (IRR = 0.53, *p* = 0.030). This significant effect disappears (*p* = 0.080; 2013–2016) over time, indicating that accredited laboratories have a better implementation procedure (Table 3).

**Table 3:** Statistical results for the association between the EQA results (EQA score, analysis errors and technical failures) and the accreditation status

	EQA score		Analysis errors		Technical failures	
	Odds ratio	*p*-value	IRR	*p*-value	IRR	*p*-value
Colon EQA scheme 2010–2016 (all laboratories), *n* = 691						
Accreditation: yes/no	1.55	0.097	0.66	0.054	1.85	**0.036**
*KRAS* accreditation (global test)	NA	NA	NA	NA	NA	0.111
Colon EQA scheme 2013–2016 (*RAS* testing laboratories), *n* = 347						
Accreditation: yes/no	1.56	0.151	0.62	0.080	1.67	0.147
Lung *EGFR* EQA scheme 2012–2016, *n* = 453						
Accreditation: yes/no	1.72	**0.018**	0.55	**0.002**	1.22	0.422
*EGFR* accreditation (global test)	NA	**0.028**	NA	**0.007**	NA	NA
Gene-specific versus none	2.62	**0.013**	0.47	**0.030**	NA	NA
Laboratory accreditation versus gene-specific	0.56	0.157	1.21	0.598	NA	NA
Laboratory accreditation versus none	1.47	0.118	0.57	**0.006**	NA	NA
Lung *ALK* EQA scheme 2012–2016, *n* = 480 (FISH) and *n* = 363 (IHC)						
FISH: Accreditation: yes/no	1.48	0.115	0.58	0.077	0.77	0.368
IHC: Accreditation: yes/no	1.86	0.056	0.88	0.662	0.21	**0.009**
*ALK* accreditation (global test)	NA	NA	NA	NA		**0.034**
Gene-specific versus none	NA	NA	NA	NA	0.24	0.118
Laboratory accreditation versus gene-specific	NA	NA	NA	NA	0.81	0.831
Laboratory accreditation versus none	NA	NA	NA	NA	0.20	**0.018**
Lung *ROS1* EQA scheme 2014–2015, *n* = 124 (FISH) and *n* = 62 (IHC)						
FISH: Accreditation: yes/no	0.62	0.192	0.90	0.823	2.37	**0.031**
IHC: Accreditation: yes/no	1.69	0.417	0.90	0.810	Only 1 failure; analysis not possible	
*ROS1* accreditation (global test)	NA	NA	NA	NA		**0.032**
Gene-specific versus none	NA	NA	NA	NA	4.22	**0.017**
Gene-specific versus laboratory accreditation	NA	NA	NA	NA	1.96	0.268
Laboratory accreditation versus none	NA	NA	NA	NA	2.15	0.067

Results are shown for accreditation in general (yes/no) as well as gene-specific accreditation status in three categories. Global test indicates a difference between the three accreditation categories: gene-specific accreditation (KRAS or NRAS), no accreditation and laboratory accreditation (no gene-specific accreditation). RAS testing laboratories included those laboratories that tested KRAS and NRAS genes

*EQA* external quality assessment, *IRR* Incidence rate ratio, *NA* not applicable

The number of years that a laboratory was accredited has no effect on the EQA result (*p* = 0.152) or the percentage of analysis errors (*p* = 0.871) for *NRAS* and *KRAS* testing laboratories between 2012 and 2014.

For *EGFR* testing (2012–2016), the total number of observations for accreditation characteristic was 453 (Table 2). An association was found between accreditation and successful EQA scores (*p* = 0.018) (score ≥ 90%), and between accreditation and fewer analysis errors (*p* = 0.002). The percentage of technical errors showed no association with the accreditation status (*p* = 0.422) (Table 3).

The EQA schemes for rearrangement analysis included the *ALK* (2012–2015) and the *ROS1 (*2014–2015) biomarker (Table 1). For rearrangement and expression analysis of *ALK* and *ROS1* genes by FISH or IHC, there was no effect between the EQA score or the percentage of analysis errors and the accreditation status (Table 3). However, accreditation is associated with less technical failures for ALK IHC (*p* = 0.009) and more technical failures for *ROS1* FISH (*p* = 0.031). Pairwise comparison of different categories showed evidence that the coverage of *ALK* is not specifically required in the scope (Table 3), however, for *ROS1* FISH, *ROS1*-specific accreditation is important (*p* = 0.017).

**Fig. 1:**
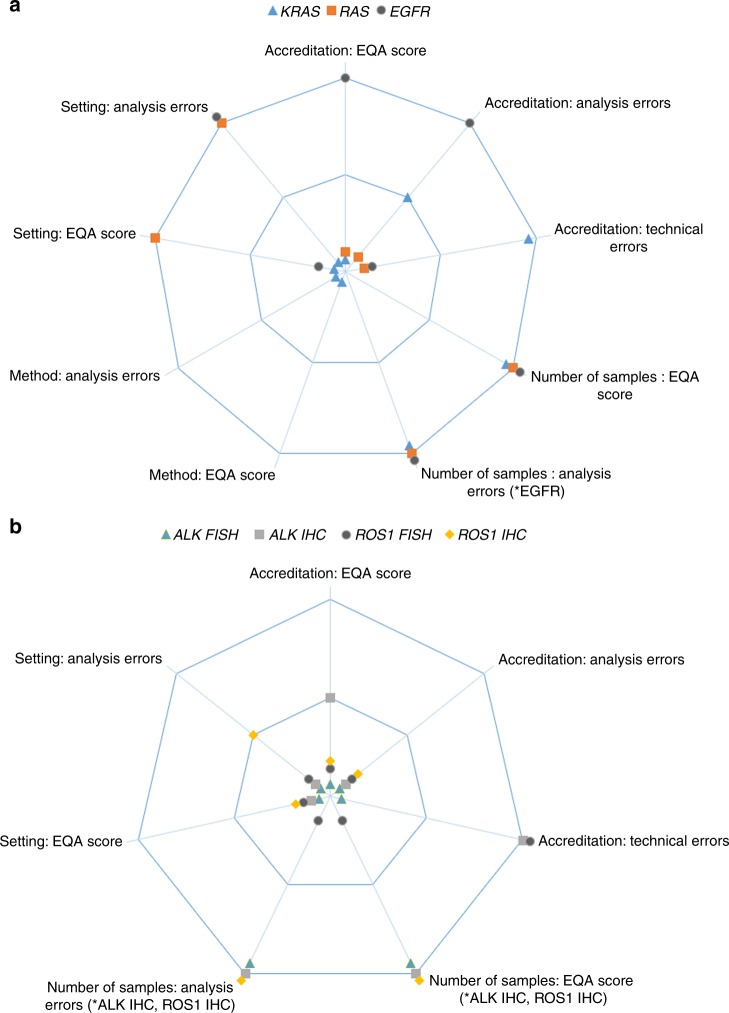
Overview of the tested characteristics. The outer line indicates the significant results (*p* < 0.05). The inner line shows the significance level of 0.05. All markers in the centre of the figure showed no significant result. **a** Variant analysis schemes. **b** Rearrangement analysis schemes. *Either the characteristic as a categorical variable or as an ordinal variable gave significant results. Analysis errors included false positive (reported variant/rearrangement/expression in a wild-type sample), false negative (wildtype reported in a tumour containing a variant/rearrangement/expression) or wrongly reported variants (correct outcome, but wrongly reported variant)

Figure 1 shows the statistical results of different characteristics as an illustrative summary of the study

### Setting

There is no evidence of an association between the setting of an institute and its EQA score or percentage of analysis errors in *KRAS* of *NRAS* testing (*n* = 711). For *RAS* analysis between 2013 and 2016 (*n* = 348), there is a significant higher probability to obtain a successful EQA score (≥90%) in the setting of university and research versus hospitals and (private) laboratories without the research incentive (*p* = 0.010 and *p* = 0.037, respectively). University and research settings were associated with less analysis errors than hospitals and (private) laboratories, and an industry setting showed less analysis errors compared to hospitals and (private) laboratories (*p* = 0.013 and *p* = 0.012, respectively). In *EGFR* testing, (*n* = 460) no association with the EQA scores was found, however, less analysis errors were made in a university and research background, compared to (private) laboratories and industry laboratories (*p* = 0.016 and *p* = 0.012, respectively).

For the rearrangement analyses, (*n* = 494, *n* = 368, *n* = 124 and *n* = 61, for *ALK* FISH, ALK IHC, *ROS1* FISH and ROS1 IHC, respectively) (Table 2), no significant associations were identified.

### Experience with oncology biomarker testing

Table 4 shows an increasing probability to have a successful EQA score with increasing number of samples tested per year for *KRAS*, *NRAS* or *EGFR* (*p* = 0.002; *p* = 0.009; *p* = 0.009, respectively). Similar results were obtained for the association with the percentage of analysis errors, except for the number of samples tested per year for *EGFR* (Table 4). In case the number of samples was considered as an ordinal variable, we observed a decreasing number of analysis errors with increasing category of the number of samples. For *KRAS*, an IRR of 0.72 indicates a decrease of 28% of genotype mistakes for every one-level increase, for example from <10 samples tested to 10–99 or from 10–99 to 100–249 samples tested. With number of samples as categorical variable, the number of analysis errors is significantly lower in laboratories that test 10–99 samples compared with laboratories that test more than 99 samples, both for *KRAS* and *NRAS*.

**Table 4:** Statistical results for the association between the EQA results (EQA score, analysis errors) and the number of samples tested per year

	EQA score		Analysis errors	
	Odds ratio	*p*-value	IRR	*p*-value
Colon EQA scheme 2010–2016 (all laboratories), *n* = 688				
*KRAS* categorical (global test)		**0.034**		**0.002**
<10 versus more than 10 samples	0.42	0.067	1.68	0.172
10–99 samples versus more than 99	0.41	**0.003**	2.23	**<0.001**
100–249 samples versus more than 249	0.69	0.256	1.55	**0.038**
*KRAS* ordinal: +1 level	1.47	**0.002**	0.72	**<0.001**
Colon EQA scheme 2013–2016 (*RAS* laboratories), *n* = 333				
*NRAS* categorical (global test)		**0.013**		**<0.001**
<10 versus more than 10 samples	0.44	0.170	1.88	0.2043
10–99 samples versus more than 99	0.22	**0.004**	3.61	**<0.001**
100–249 samples versus more than 249	0.51	0.419	1.94	0.0938
*NRAS* ordinal: +1 level	1.58	**0.009**	0.64	**<0.001**
Lung *EGFR* EQA scheme 2012–2016, *n* = 457				
*EGFR* categorical (global test)		**0.015**		**0.041**
<10 versus more than 10 samples	0.43	**0.044**	1.88	**0.005**
10–99 samples versus more than 99	0.74	0.252	1.13	0.543
100–249 samples versus more than 249	0.52	**0.010**	1.16	0.429
*EGFR* ordinal: +1 level	1.30	**0.009**	0.92	0.085
Lung *ALK* EQA scheme 2012–2016, *n* = 384 (FISH) and *n* = 305 (IHC)				
*ALK* FISH categorical (global test)		**<0.001**		**0.018**
<10 versus more than 10 samples	0.19	**<0.001**	3.03	**0.002**
10–99 samples versus more than 99	0.40	**0.013**	2.18	0.071
100–249 samples versus more than 249	0.61	0.277	1.48	0.529
*ALK* FISH ordinal: +1 level	1.79	**<0.001**	0.68	**0.005**
*ALK* IHC categorical (global test)		**0.022**		**0.003**
<10 versus more than 10 samples	0.39	**0.032**	2.78	**0.009**
10–99 samples versus more than 99	0.83	0.647	0.70	0.325
100–249 samples versus more than 249	1.91	0.184	0.80	0.641
*ALK* IHC ordinal: +1 level	1.19	0.269	0.92	0.595
Lung *ROS1* EQA scheme 2014–2015, *n* = 122 (FISH) and *n* = 62 (IHC)				
*ROS1* FISH categorical (global test)		0.865		0.616
*ROS1* FISH ordinal: +1 level	1.01	0.937	0.97	0.890
*ROS1* IHC categorical (global test)		**0.038**		0.234
<10 versus more than 10 samples	0.47	0.449	NA	NA
10–99 samples versus more than 99	10.47	**0.006**	NA	NA
*ROS1* IHC ordinal: +1 level	1.44	**0.306**	1.36	**0.031**

For categorical variables, the global test shows the difference between the categories: <10, 10–99, 100–249, 250–499, 500–999, ≥1000. Post hoc comparisons are only performed when the global *p*-value is significant. RAS testing laboratories included those laboratories that tested KRAS and NRAS genes

*EQA* external quality assessment, *IRR* incidence rate ratio, *NA* not applicable

For Lung *ALK* FISH, a higher number of samples tested per year was associated to an improved EQA score and a decreased percentage of analysis errors (Table 4). For an increase in level of the samples tested per year, 32% fewer analysis errors were made with *ALK* FISH analysis (IRR = 0.68, *p* = 0.005). This is in contrast with results for *ROS1* rearrangement testing, where no association could be observed between the *ROS1* FISH EQA scores or analysis errors and the number of samples tested for *ROS1*.

For IHC tests, there was evidence of variation between EQA scores and the categories of number of samples tested, however, there is no conclusive evidence that the EQA score improved with an increasing number of samples tested per year. The percentage of analysis errors showed a difference between the number of *ALK* IHC testing categories, but no conclusive results could be made that less analysis errors were made when more samples were tested for *ALK*. For ROS1 IHC, however, a 36% increase of ROS1 IHC analysis errors was seen for every increase in the level of number of samples tested per year.

**Fig. 2:**
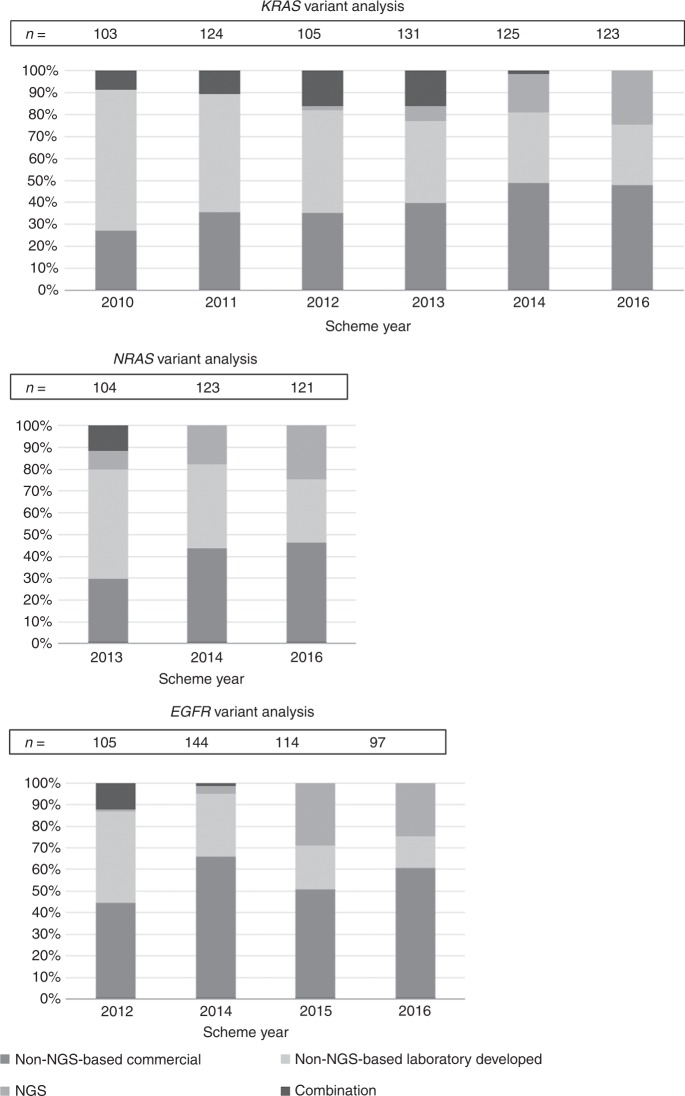
Longitudinal overview of the distribution of techniques in different EQA schemes for (**a**) (KRAS variant analysis, **b** NRAS variant analysis and **c** EGFR variant analysis. NGS next-generation sequencing, Commercial includes non-NGS-based commercial kits, laboratory includes developed non-NGS-based laboratory developed

In addition to the number of samples tested in a laboratory, changing a routinely applied method can also influence the experience of a laboratory with a specific biomarker. The methods used in molecular pathology laboratories vary greatly. Figure 2 gives an overview of the evolution of the distribution of different types of methods over the years for the *RAS* and *EGFR* biomarkers. In general, less LDTs were used over the years, while non-NGS-based commercial methods and NGS-based techniques gained more weight. At the time that a new predictive biomarker was introduced in the routine, a limited number of non-NGS-based commercial test kits was available. The implementation of a new method for *KRAS* variant analysis (2010–2014) showed no significant association with the EQA score or the number of analysis errors (*p* = 0.471, *p* = 0.871, respectively). As not enough data were available on other biomarkers, no further analyses could be performed.

## Discussion

The changing era of personalised medicine challenges laboratories to offer quality-assured biomarker testing and maintain a high performance level for their tests. Different laboratory characteristics are accepted as important tools for increased quality assurance, but the question remains whether these should be mandatory or not. This study investigated four characteristics in laboratories testing for biomarkers in NSCLC and mCRC: accreditation status of a laboratory, their setting, the number of samples tested each year and the change in used methods.

Less analysis errors were observed in accredited laboratories in the early stages of biomarker introduction. This was demonstrated by the positive effect for NRAS variant analysis, which is a new biomarker introduced in laboratories in 2013, and EGFR variant analysis.^5,6^ These two biomarkers (EGFR and NRAS) are essential for therapy decision-making and will challenge the laboratories because of the need of fast introduction in the routine or the large spectrum of variants that needs to be tested.^4,31^ The inclusion of the newly introduced biomarker in the accreditation scope is not essential, as laboratory accreditation is intense and laborious and stimulates the laboratory in a significantly positive way.^32^ Implementation of new tests in the accredited laboratories should follow strict standards, including the introduction of well-planned and validated standard operating procedures. This can be seen as a drawback of accreditation, as it could lead to postponing the introduction of new developments. However, in the context of biomarkers, laboratories are forced to introduce these changes, as they become mandatory before therapy decision. In that case, accreditation forms a welcomed basis during introduction.

Once the biomarker is successfully implemented in the laboratory, the association between good performance and laboratory accreditation disappears. While accredited laboratories have addressed the difficulties during the extensive validation procedure, laboratories without accreditation have gained experience during the routine execution of biomarker testing and perform as well as accredited laboratories in the long term. The positive association continues for the *EGFR* biomarker. The LUNG *EGFR* EQA scheme remains a challenging scheme in the long term because of the use of different types of sample material (cell line material versus resection specimens), which was not always included by the laboratories in their initial validation and the large spectrum of variants.

Nevertheless, in the long term, the effect of accreditation seems to gain more influence again, as there is a trend towards less analysis and technical errors in accredited laboratories for the routinely used *KRAS* biomarker. A laboratory with accreditation must remain attentive and should be able to guarantee a high quality assurance, even after many years of routine analysis.

The hypothesis that accreditation status of a laboratory has a positive impact on the correctness of results also has a role in reporting conclusive results for gene rearrangement and expression analyses. Accredited laboratories were more careful in reporting conclusive results for *ROS1* FISH analysis, but no association could be found with *ALK* FISH. At the time of the analyses, in 2012, *ROS1* was a recently discovered biomarker, not yet included in any European label for anti-EGFR tyrosine kinase inhibitors (TKI).^33,34^ This might be an explanation for the contradiction.^35^ Once the *ROS1* biomarker becomes a well-known relevant biomarker, the data should be re-analysed to observe whether the significant effect disappears after few years, similar as with the variant analysis biomarkers. Accredited laboratories reported fewer technical errors for ALK IHC, while there was no association between the percentage of technical failures and accreditation for *ALK* FISH rearrangement analysis. This test is for most laboratories technically more demanding and difficult to interpret than IHC analysis. Laboratories, independent of their accreditation status, may introduce this test only after profound training of their personnel, such that they are able to give valid and conclusive interpretations. At the time of the analysis, this analysis could not be repeated for ROS1 IHC, as no data were available.

Laboratories that work within a setting of university and research made less analysis errors in variant analysis testing than laboratories in another setting. It appears that university and research laboratories have more extensive experience and are used to innovative methodology, while private and industry laboratories are more stimulated to work in a cost-effective way. It is expected that this significant result will disappear within a few years, when new or complex biomarkers are fully integrated into the routine workflow of the laboratories, like what was observed with the accreditation indicator. There were no signs that these two indicators were linked to each other, as the university and research laboratories have an almost equal distribution of accreditation or no accreditation.

The number of samples analysed per year for *RAS* and *EGFR* can be considered as an influential indicator for good laboratory performance. There is no evidence that this indicator was linked to the previously discussed indicators of accreditation and setting. *RAS-*testing of more than 99 samples, compared to 10–99 samples gave a higher probability of a better EQA score and less analysis errors. Using these results advocates that centralised biomarker testing improves quality. Development of a centralised approach to testing of predictive markers should be promoted soon. Such a centralised approach will increase the experience of the laboratory and will be beneficial for the performance of laboratories performing variant analyses of oncology biomarkers.

There is evidence of an improved EQA FISH performance for a larger number of samples tested per year for *ALK*, which is absent for analysis of *ROS1* rearrangements (Table 4). *ROS1* was a new biomarker that was introduced in the laboratory before it became a mandatory biomarker. In this case, the number of samples tested per year seems to have no influence on the EQA results of FISH analysis. Once the biomarker becomes clinically relevant and routinely used, enough samples should be tested per year to maintain the high level of experience as every increase in the level of number of samples tested implies a decrease in analysis errors of 32% (Table 4). It should be taken into account that less data were available for *ROS1* analysis compared to *ALK* analysis so this conclusion should be confirmed in the future.

FISH analysis, including the interpretation, is technically more difficult compared to a more straightforward IHC analysis. This can explain why no linear association was found between ALK IHC analysis and EQA results. Regarding ROS1 IHC analysis, there was even an increase in false positive and false negative results when more than 99 *ROS1* samples were analysed with IHC per year compared to 10–99 samples. Also, IHC analysis mainly depends on antibody sensitivity while FISH analysis requires interpretation by an experienced operator. It was expected that more experience with FISH samples is thus needed during the introduction of a new biomarker. However, this cannot be demonstrated in this study.

The results show an increased use of NGS-based methods and a decreased use of non-NGS-based LDTs for variant analysis (Fig. 2). Considering the broader spectrum of relevant biomarkers, this is a logical evolution in molecular pathology testing. At the time of introduction of the *NRAS* biomarker, the availability of non-NGS-based commercial test kits for *NRAS* variant analysis was limited.^14^ There was an extensive use of non-NGS-based LDTs for *NRAS* analysis in the Colon EQA scheme of 2013, which was significantly higher than for *KRAS* variant analysis (p = 0.047). Over the following years, the ratio changed as more non-NGS-based commercial *NRAS* test kits became available. The change in methods does not seem to influence the EQA results. Consequently, this characteristic cannot be an indicator of good quality assurance of biomarker testing.

## Conclusion

The hypothesis to impose accreditation of laboratories, to centralise biomarker testing or to test within a setting of university and research was supported by this study. Results of EQA schemes in mCRC and NSCLC show that improvement of performance in oncology biomarker testing is associated with three of the four tested characteristics. First, laboratory accreditation is needed to ensure high quality and reliable implementation of new diagnostic molecular biomarkers. Second, university and research laboratories reach more optimal results. Third, larger number of samples tested per year proved to be an indicator for good EQA results, implying more centralisation of biomarker testing to reach sufficiently high volumes. Finally, changes in applied methods do not influence laboratory performance.

### Data availability

Data supporting the results can be obtained upon request.
